# Sensorineural Hearing Loss After Balloon Eustachian Tube Dilatation

**DOI:** 10.3389/fsurg.2021.615360

**Published:** 2021-03-05

**Authors:** Ingo Todt, Felix Oppel, Holger Sudhoff

**Affiliations:** Medical School OWL, Bielefeld University, Klinikum Bielefeld Mitte, Bielefeld, Germany

**Keywords:** eustachian tube, balloon dilatation, hearing loss, complication, SNHL

## Abstract

**Objective:** Eustachian tube function is of central importance for the ventilation of the middle ear. A dysfunction can be associated with chronic otitis media, and cholesteatoma. Balloon Eustachian tube dilatation (BET) is a treatment option used to solve eustachian tube dysfunction. Although BET is widely performed, little is known about the occurrence rate of the complications associated with BET. The aim of the present study was to observe the rate of sensorineural hearing loss (SNHL) after BET.

**Methods:** We retrospectively evaluated in a chart review 1,547 patients and 2,614 procedures of BET performed in a single center between 2015 and 2019 using the Spiggle and Theis, Overath, Germany eustachian tube dilatation system.

**Results:** We observed seven cases of SNHL after BET. In two cases, the SNHL persisted, and in five cases, the SNHL was transient. In two cases of SNHL, a simultaneous tympanoplasty was performed. The overall rate of SNHL per procedure is 0.3%. The rate of permanent SNHL is 0.08%.

**Conclusion:** BET has a low rate of SNHL. Rapid middle ear pressure changes are assumed to cause BET-related hearing loss.

## Introduction

Eustachian tube dysfunction (ETD) is known to be one of the most common problems seen in ENT-clinics and has an estimated prevalence of 1–5% in adults (1, 2). Up to now, the function of the Eustachian tube is considered to be highly complex. ETD can cause clinical symptoms such as intermittent or chronic ear pressure, aural fullness, tinnitus, ear noises (such as cracking and popping), discomfort, numbness of the ear, hearing loss, and autophony (3, 4). The physiological functions of the Eustachian tube include the ventilation of the middle ear, mucocilliary clearance of secretions, pressure equalization, protection from sounds, and pathogens ascending from the nasopharynx. As a long-term complication, ETD can lead to pathologic changes in the tympanic membrane with adhesive processes, retraction pockets, and chronic otitis media with effusion.

Regarding the treatment of ETD, multiple techniques have been used like treatment with nasal xylometazoline and grummets. One focused on solving obstructions at the level of the isthmus within the Eustachian tube using laser Eustachian tuboplasty (LETP), developed by Kujawski in 1997. A technique using a carbon dioxide or 980-nm diode laser to vaporize the mucosa and cartilage of the luminal posterior wall of the pharyngeal ostium (5).

The balloon dilation of the Eustachian tube, developed by Sudhoff (6), presents a non-cutting surgical procedure for treating ETD. In this procedure, an inflatable balloon catheter is placed into the Eustachian tube through the nasopharyngeal ostium under an endoscopic view. The balloon catheter is then inflated with saline to 10 bars and stays positioned in the Eustachian tube for 2 min. In the following, the catheter is deflated and removed.

The indication of performing a BET based on clinical findings like fullness of the ear, pain in specific situations (air flights, driving), recurrent OM or cholesteatoma with negative valsalva testing, recurrent sero- and mucotympanon after paracentesis or tubes. Testings like negative valsalva testing, questionnaires and tubomanometry. Hearing threshold was not usefull for the indication for BET. For further information see Tysome (7).

An evaluation of more than 622 patients BETs from 1,076 BETs 2 months after the performed procedure showed a high rate of improvement in patients in terms of eustachian tube score (8). Through a 9-year evaluation, the balloon dilatation of the Eustachian tube proved to be an easy-to-perform, low-risk procedure (8). Also, in a randomized controlled study, the long-term evaluation results of patients who underwent BET revealed a high rate of the definite improvement of their clinical symptoms (9). Based on the study of Poe et al., BET is a validated treatment for ETD (10) in selected cases.

Concerning the occurrence of minor complications, transient adverse events are reported with a rate of 3% (11), including nose bleeding and hematotympanum. The rate of cervicofacial and mediastinal emphysema as major complications was calculated as 0.27% in a multicenter study with 3,670 BET procedures (12). The information of a negative effect on the inner ear function is of central importance for a procedure which is focussed on improving the middle ventilation and function. So far there is no study looking at this specific concern.

The occurrence of SNHL has been discussed and anecdotally described as a possible major complication. The aim of this study was to evaluate the rate of SNHL related to the BET procedure.

## Materials and Methods

We performed a retrospective chart review of all performed BET procedures (*N* = 2,614) between 2015 and 2019 in 1,547 patients in the Department of Otolaryngology, Head and Neck Surgery, Campus Mitte, Bielefeld University.

All procedures were performed with the BET dilatation system transnasal with the insertion tool by Spiggle and Theis, Overath, Germany. In all cases the dilatation was an inpatient procedure and a hearing threshold, tympanometry, tubomanometry and Valsalva were performed. Indication for BET was in most of the cases an obliterative ETD and a negative Valsalva (7). The procedure was performed independent from the preoperative SNHL. Six hours and at the morning of the next day after the procedure the patients were asked for any change in their subjective hearing. If so, a pure tone audiogram was performed even at the following days. If a sensorineural hearing (BC) loss was observed a high dose prednisolone i.v. application for 3 days was given (250 mg each day). If a hearing loss persists an iv. steroid application over 10 days was given. Tympanometry, tubomanometry and Valsalva follow up data were not collected after the occurrence of SNHL in this group.

The data set of all cases was scanned for SNHL and an accompanied change of the hearing threshold. Individual demographic data are added to the Table 1. Individual PTA preoperative and post operative are given in Table 2. Anonymous data analyses were performed with SPSS Version 24.

**Table 1:** Individual data of patients with hearing loss after BET, Tymp. indicates tympanometry results, n.m. means not measured; Valsalva result, and tympanomtry results in mmHg opening pressure (50 neg, means no opening could be observed).

**Pat**.	**Sex**	**Age**	**Surgery year**	**Concurrent surgery**	**Side**	**Recovery/permanent**	**Additional information**	**Tymp**.	**Valsalva**	**Tubo**
1	f	46	2016		left	recovery	emphysema	reg	negative	30 pos
2	f	58	2017		right	recovery		flat	negative	30 pos
3	f	70	2018	paracentesis	left	recovery		flat	negative	50 neg
4	m	65	2018	paracentesis	right	permanent		n.m.	negative	50 neg
5	f	55	2018		right	permanent	med.SNHL	reg	negative	30 pos
6	m	64	2019	tympanoplasty	right	recovery		n.m.	negative	50 neg
7	m	77	2019	tympanoplasty	left	recovery		n.m.	negative	50 neg.

**Table 2:** Individual PTA data (BC).

**Pat**.	**Pre OP** **in kHz**	**0.5**	**1**	**2**	**3**	**4**	**Mean**	**Post OP** **in kHz**	**0.5**	**1**	**2**	**3**	**4**	**Mean**	**dB loss**
1		15	20	20	20	20	19		30	40	40	40	40	38	19
2		5	5	10	10	10	8		25	20	25	30	25	25	17
3		15	15	20	25	30	21		15	20	40	45	50	34	13
4		30	45	50	60	60	49		75	70	55	65	80	69	20
5		20	25	30	30	30	27		10	45	55	50	50	42	15
6		30	40	45	45	40	40		35	45	65	35	40	44	4
7		25	20	35	45	45	34		35	35	40	65	45	44	10

All procedures were performed in accordance with the current Helsinki declaration and amendments or ethical standards.

## Results

**Figure 1:**
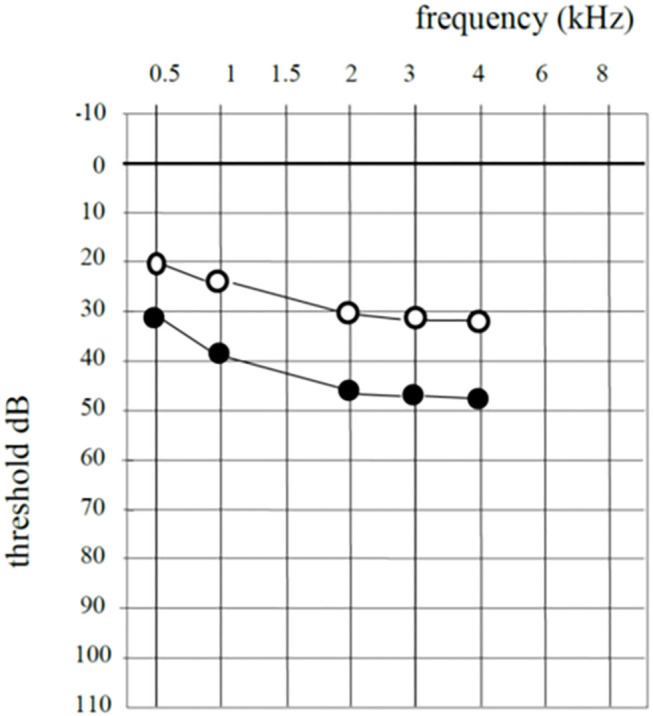
Mean hearing threshold in cohort group.

**Figure 2:**
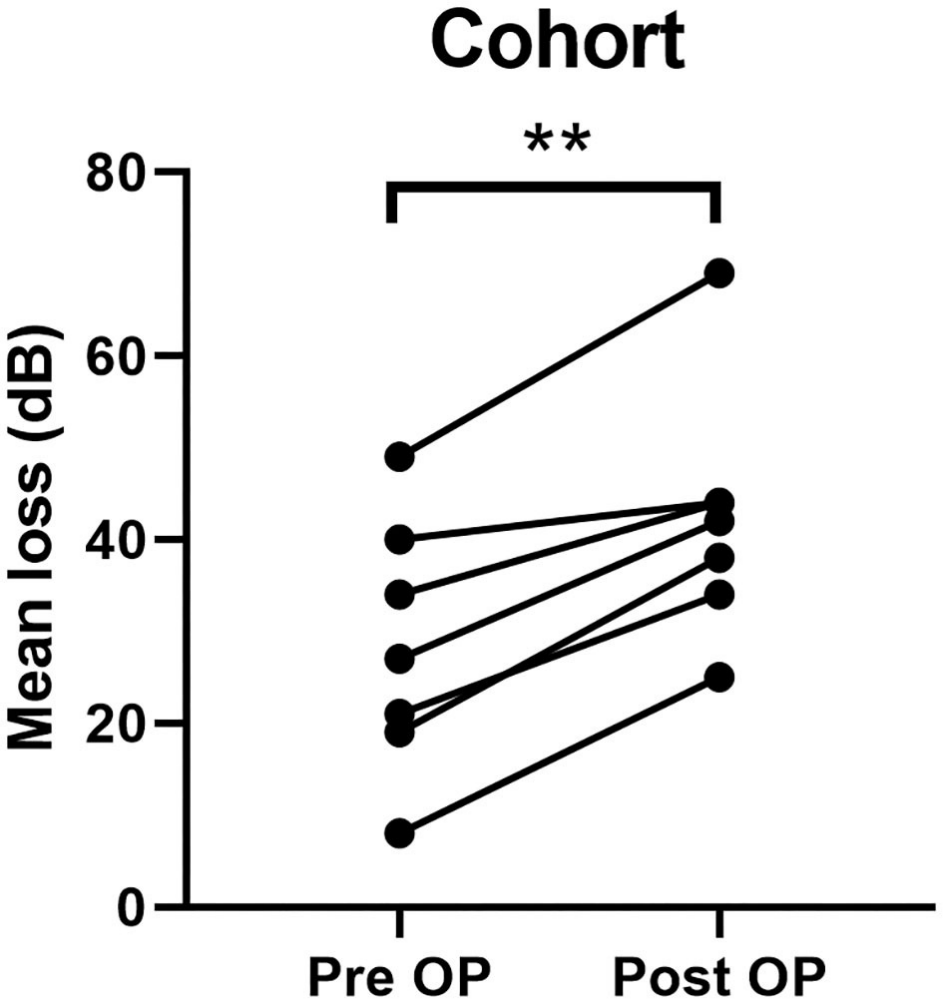
Statistical analysis of cohort group. **indicates a significant difference.

SNHL was observed in seven cases. In all cases hearing loss was subjectively observed 6 h after the procedure. In all cases, intravenous steroids were administered. In five patients, SNHL recovered. In all cases of recovery hearing threshold removed to the preoperative level within 3 days. In two patients, SNHL remained unchanged. Table 1 shows the individual data. Table 2 shows the individual SNHL pre and postoperative (Figure 1). Mean dB loss was 14 dB which is a significant difference to the pre OP threshold. Wilcoxon matched-pairs signed rank test, with 95% confidence interval, ^**^ ≤ 0.01). The exact *p*-value is 0.0078 (Figure 2).

Mean dB loss for the transient cases was 12.6 dB. Mean dB loss for the permanent cases was 17.5. In cases of tympanoplasty the BET was performed before the tympanoplasty and in cases of paracentesis the BET was performed before. In both of these tympanoplasty, SNHL was restored. The overall rate of hearing loss per procedure is 0.3%. The rate of permanent hearing loss is 0.08%.

## Discussion

A regular tubal function is of central importance to the ossicular energy transfer from the middle ear to the inner ear. One therapeutic option for cases of obliterative tubal dysfunction is BET. BET, performed since 2010, has a described success rate of up to 83% (8). Recent studies showed a positive long-term effect (8) and could validate the procedure against a conservative treatment as superior (10).

The underlying functional mechanism is assumed to be multi-factorial. It is discussed that the process involves microfractures of the cartilage (2), the extraction of mucus; and the removal of adhesions, fibrous strands, and biofilms. In addition, the presence of an enlarged intratubal lumen after the procedure is undoubtful.

In addition to being a pure mechanical interaction, pressure generation is a mode of action.

The rate of minor complications, such as nose bleeding, or hemotympanum, has been described to be 3%. Major complications, such as emphysema, have been described to occur in 0.27% of cases (12). In all patients, emphysema has not led to lasting functional deficiencies. The assumed underlying mechanism is the rupture of the mucosa during the insertion (12). Related to this, a design change at the tip of the Spiggle and Theis catheter was performed 2018. After this design modification, no emphysema has occurred in our department. So far even no case of SNHL occurred.

The rate of SNHL in this study is 0.3%. The underlying mechanism might be explained by middle ear pressure changes, because the regular maximum pressure change during the procedure is 180 daPa (13.8 mmHG) (13) and an irregular fast removal of the catheter has been shown to be up to 80 mmHg (14). This value compared to the described rupture force of the round window for cats (6–66 mmHG) (15), lets us assume that a pressure-related rupture seems possible. Human data are lacking to our knowledge. Another mechanism might be a fast intracochlear pressure change-generated effect above the inner ear's ability to compensate static pressure changes. Even the individually increased fragility of the round window or micro lesions might play a role.

Another possible mechanism might be the insertion of the catheter without the usage of the insertion tool, which defines the depth of insertion. This handling might lead to an exaggerated insertion depth of the balloon under the long process of the incus, resulting in a dislocation of the stapes into the vestibulum and joint disruption. Because no conductive component was observed in our cases, this can be ruled out in our cases.

In our series, two cases of hearing loss occurred when tympanoplasty and BET were performed in combination. In these cases, we cannot rule out that the SNHL was not associated to BET but rather to the surgical procedure of tympanoplasty [(16); 1.2% hearing loss]. If we follow the hypothesis of pressure related SNHL this seems reasonable.

For the underlying mechanism of SNHL with recovery, we assume that a non-compensated static pressure changes or a round window micro lesion was responsible.

For the cases of the non-recovery of the threshold, a rupture of the round window is assumed to be the cause. Computed tomography scanning was not performed in our cases of hearing loss possibly showing air in the cochlea as a consequence of a round window rupture.

The careful and slow insertion tool guided insertion and removal of the catheter seems to be the safest way for the prevention of BET-related SNHL. In cases of mucotympanon paracentesis, should be performed before the BET procedure.

Additionally, we cannot fully rule out cases of sub clinical low frequency SNHL, which are perceived as aural fullness.

This is to the best of our knowledge the first study describing cases of SNHL related to the BET procedure. This is of high importance since it quantifies for the first time the rate of this especially for otologist important complication. The reason for this first description might be related to the high number of patients and on the other hand low rate of SNHL. The rate of unkown sudden sensorineural hearing loss (ISSNHL) in the german population of 0.16% (17) underlines the assessment of a low rate of SNHL after and caused by BET.

Limitation of this study is the retrospective character. Since not all cases of BET are postoperatively regularly evaluated for SNHL, cases of subclinical low frequency hearing loss can be missed. Even an objective evaluation by ABR or OAE was not performed at this acute stage.

## Conclusion

The BET has a low rate of SNHL. Rapid middle ear pressure changes are assumed to cause BET-related hearing loss.

## Data Availability Statement

The original contributions presented in the study are included in the article/supplementary material, further inquiries can be directed to the corresponding author/s.

## Ethics Statement

The studies involving human participants were reviewed and approved by Institutional Review Board Klinikum Bielefeld. The patients/participants provided their written informed consent to participate in this study. Written informed consent was obtained from the individual(s) for the publication of any potentially identifiable images or data included in this article.

## Author Contributions

IT: writing, design, idea, data collection, final approval, and accountable for all aspects. FO: co-writing and statistics, final approval, and accountable for all aspects. HS: design, writing and discussion, final approval, and accountable for all aspects. All authors contributed to the article and approved the submitted version.

## Conflict of Interest

HS received research grants and speaker payments by Spiggle and Theis. The remaining authors declare that the research was conducted in the absence of any commercial or financial relationships that could be construed as a potential conflict of interest.
